# Supplementary material for: Exploring the Effect of Ethnicity on Chronic Orofacial Pain: A Comparative Study of Jewish and Arab Israeli Patients
Source: Healthcare (Basel). 2023 Jul 8;11(14):1984. doi: 10.3390/healthcare11141984 (PMC10379038; doi:10.3390/healthcare11141984)
Supplement: Supplementary file 1 [file healthcare-11-01984-s001.zip › healthcare-2434703-supplementary.pdf]

## Figure S1

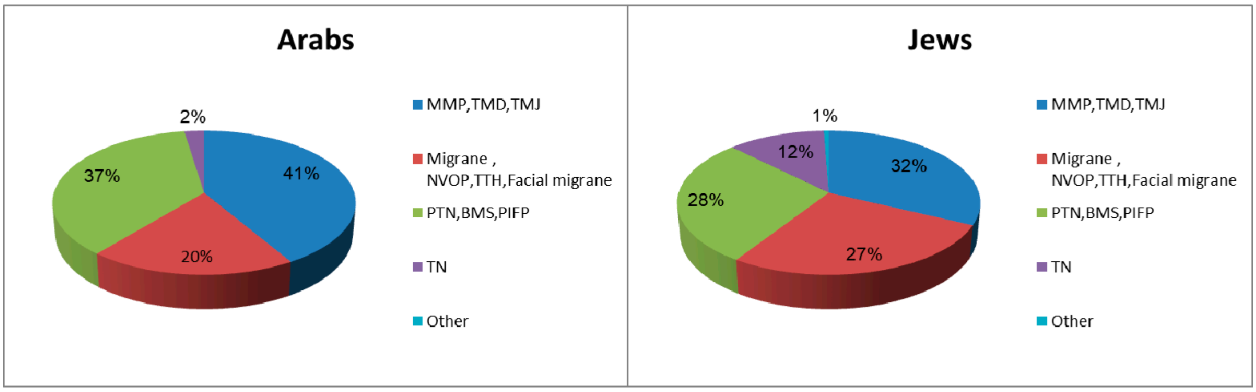


Specific Pain diagnosis distribution in each ethnic group:

-Musculoskeletal group - MMP - Masticatory Myofascial Pain, TMD - Temporomandibular disorder, TMJ - Solely Temporomandibular joint origin; -Neurovascular group - Migraine including NVOP -Neurovascular orofacial pain (orofacial migraine) and facial Migraine; -Neuropathic Group - PTN - Post Traumatic Neuropathy, PIFP – Persistent idiopathic facial pain, BMS – Burning Mouth Syndrome; -TN - Trigeminal neuralgia (Neurological Disorder) ;
